# Intestinal Microbiota: The Driving Force behind Advances in Cancer Immunotherapy

**DOI:** 10.3390/cancers14194796

**Published:** 2022-09-30

**Authors:** Zhujiang Dai, Jihong Fu, Xiang Peng, Dong Tang, Jinglue Song

**Affiliations:** 1 Department of Colorectal Surgery, Xinhua Hospital, Shanghai Jiaotong University School of Medicine, Shanghai 200092, China; 2 Shanghai Colorectal Cancer Research Center, Shanghai 200092, China; 3 Department of General Surgery, Institute of General Surgery, Northern Jiangsu Province Hospital, Clinical Medical College, Yangzhou University, Yangzhou 225001, China

**Keywords:** microbiota, immunotherapy, metabolism, immune checkpoint inhibitors, FMT

## Abstract

**Simple Summary:**

Despite the great achievements of cancer immunotherapy in a variety of tumors, tumor heterogeneity and drug resistance still plague patients and clinical researchers. In particular, the occurrence of immune-related adverse events forces patients to discontinue cancer immunotherapy. Therefore, it is urgent to optimize cancer immunotherapy and improve the efficacy of immunotherapy. With the iteration of sequencing technology, the microbiome, as the second set of genomes in the body, has been proven to be involved in immunity and metabolism. More and more studies are gradually shifting the perspective to the intestinal microbiota and cancer immunotherapy. The intestinal microbiota reactivates and modulates immune cells in immunotherapy and is expected to become a biomarker for predicting immune efficacy. Targeting to improve the intestinal microbiota can enhance anti-tumor immunity. This advantage is beneficial to control related adverse symptoms and expand the beneficiary population of cancer immunotherapy. This finding can help clinicians comprehensively evaluate the effect of tumor screening and tumor treatment. Therefore, the innovative combination of gut microbiota and cancer immunotherapy is expected to be an active strategy to enhance individualized immune responses.

**Abstract:**

In recent years, cancer immunotherapy has become a breakthrough method to solve solid tumors. It uses immune checkpoint inhibitors to interfere with tumor immune escape to coordinate anti-tumor therapy. However, immunotherapy has an individualized response rate. Moreover, immune-related adverse events and drug resistance are still urgent issues that need to be resolved, which may be attributed to the immune imbalance caused by immune checkpoint inhibitors. Microbiome research has fully revealed the metabolic-immune interaction relationship between the microbiome and the host. Surprisingly, sequencing technology further proved that intestinal microbiota could effectively intervene in tumor immunotherapy and reduce the incidence of adverse events. Therefore, cancer immunotherapy under the intervention of intestinal microbiota has innovatively broadened the anti-tumor landscape and is expected to become an active strategy to enhance individualized responses.

## 1. Introduction

Currently, immunotherapy is considered one of the most cutting-edge technological revolutions in the field of clinical oncology. Immune checkpoint inhibitors (ICIs) (such as anti-programmed death receptor 1/programmed death ligand 1 (PD-1/PD-L1), anti-cytotoxic T lymphocyte antigen 4 (CTLA-4)) indirectly exert active and effective anti-tumor immunity by inducing suppressed T cell activation [1]. Based on a large amount of clinical and medical evidence, some ICIs have been approved by the drug regulatory agency for trials or treatments of various malignant tumors [2]. However, most patients still cannot fully benefit from ICIs, which are often accompanied by primary drug resistance or immune-related adverse events [3].

In order to improve the titer of immunotherapy, actively seeking to serve biomarkers for ICIs response and toxicity prediction has become an urgent barrier to be resolved. The iterative update of sequencing technology has opened up an emerging situation in immunotherapy research [4]. The rise of metagenomics and bioinformatics has provided a new perspective on the development of microbiology in tumorigenesis [5,6]. Additionally, more and more evidence supports the important position of the microbiome in the immune-metabolic interaction of cancer, especially in response to blocking immune checkpoints [7,8,9]. This article reviews the promising future of the gut microbiota in cancer immunotherapy, including fecal microbial transplantation (FMT), probiotics, and prebiotic preparations. The intestinal microbiota is expected to provide assistance in opening up a new pattern of anti-tumor immunotherapy.

## 2. The Amazing Therapeutic Potential of Cancer Immunotherapy

Reports about ICIs making breakthrough progress in the field of tumor treatment are increasing day by day. Currently, the most widely used ICIs in clinical trials include anti-PD-1/PD-L1 (nivolumab/pembrolizumab/durvalumab/atezolizumab) and anti-CTLA-4 (ipilimumab) [10]. The Topalian team found that nivolumab combines clinical efficacy and high safety in the treatment of various solid tumors such as melanoma [11]. The results showed that patients with advanced melanoma without BRAF mutations had significantly improved overall survival (OS) after receiving nivolumab treatment. Meanwhile, the results of the study were also approved by another phase III randomized double-blind trial [12]. In addition, Takamida et al. reviewed the efficacy of the first-line regimen containing pembrolizumab in patients with metastatic non-small cell lung cancer (NSCLC) with high PD-L1 expression (tumor proportion score (TPS) ≥ 50%) in the past three years [13]. The study clarified that the first-line pembrolizumab single-agent regimen significantly prolonged the patient’s OS and progression-free survival (PFS). Additionally, in the case of similar TPS, pembrolizumab combined with chemotherapy and monotherapy was beneficial to the survival of NSCLC patients [14]. However, another randomized controlled phase 3 trial comparing pembrolizumab with locally advanced NSCLC chemotherapy (KEYNOTE-042) revealed that the OS of the pembrolizumab group was significantly longer than that of the chemotherapy group (*p* = 0.0018) [15]. Therefore, the important position of pembrolizumab as the first-line standard therapy to treat NSCLC with high PD-L1 expression was affirmed.

Colorectal cancer (CRC) is a highly heterogeneous tumor, and its different subtypes have differentiated responses to immunotherapy [16]. CRC with high microsatellite instability (MSI-H) has high tumor mutational burden (TMB), TH1 immune infiltration, and immune checkpoint gene expression, which would have a positive effect on immunotherapy [17,18]. In MSI-H CRC, higher TMB is the objective reaction of pembrolizumab immunotherapy and the strongest biomarker of PFS [19]. However, this part of the patient accounts for 5% of the transfer of CRC patients. Microsatellite stabilization (MSS) accounts for 95% of patients with metastasis CRC and is resistant to immunotherapy [20]. High TMB MSS CRC patients have reactions to PD-1 inhibitors [21]. In addition, a prospective clinical trial (Keynote-158) found that the high TMB (TMB > 10) can effectively predict the efficacy and prognosis of Pembrolizumab [22]. This phenomenon suggests that high TMB may be used as an indicator of MSS CRC, which is conducive to the patient obtaining immunotherapy benefits. We briefly summarize the application and effect of some ICIS in solid tumors for reference (Table 1).

Currently, multiple groups of clinical trials provide evidence to support the combination of immunotherapy and chemotherapy for the treatment of a variety of malignancies. NSCLC is a pioneer in trials combining chemotherapy and immunotherapy. After observing that immunotherapy was effective in PD-L1-high (>50%) tumors and in patients treated with platinum chemotherapy, clinicians performed immunotherapy as maintenance in a phase II trial [31]. The results showed that the therapy had a significant effect on adenocarcinoma (HR 0.49; *p* < 0.001) and squamous cell carcinoma (HR 0.64; *p* < 0.001) [32]. A phase III clinical trial found that pembrolizumab combined with chemotherapy (paclitaxel/carboplatin/gemcitabine) had a significant benefit in patients with a composite positive score (CPS) ≥ 10 compared with chemotherapy alone (HR 0.65; *p* = 0.00411) [33]. A meta-analysis of chemotherapy combined with immunotherapy in metastatic triple-negative breast cancer (mTNBC) revealed that the addition of PD-1/PD-L1 blockade to chemotherapy improved PFS in patients with PD-L1-positive mTNBC [34]. Therefore, immunotherapy has great development space for the original tumor treatment system.

## 3. Combination Immunotherapy

Although ICIs show surprising therapeutic potential in tumor treatment, some patients still have difficulty benefiting from monotherapy. In order to improve the universality of immunotherapy, clinical researchers have turned their attention to combination therapy in an attempt to expand the beneficiaries of immunotherapy [35,36]. Hodi et al. evaluated a phase II randomized controlled trial of combination therapy of nivolumab and ipilimumab and monotherapy of ipilimumab for advanced melanoma [37]. Compared with ipilimumab monotherapy, the effect of combination therapy is more satisfactory [37]. Additionally, Hodi et al. added that regardless of whether BRAF is mutated or not, the first-line combination regimen or single-agent regimen can exert long-lasting clinical benefits in patients with advanced melanoma, but the combination can better improve patient survival. In order to verify whether the combination of nivolumab and ipilimumab or pembrolizumab as a chemotherapy-free first-line therapy can improve survival, Zhou et al. incorporated three randomized trials of KEYNOTE-024, KEYNOTE-042, and Checkmate 227 into the research system using the frequency method [38]. A combined meta-analysis suggested that both regimens can improve OS compared with chemotherapy (combination therapy: HR 0.82, 95% CI 0.69–0.97; monotherapy: HR 0.81, 95% CI 0.71–0.93), but only combination therapy improved PFS (combination therapy: HR 0.79, 95% CI 0.65–0.96; monotherapy: HR 1.07, 95% CI 0.94–1.21). However, combination therapy showed a higher rate of adverse events. A multicenter, retrospective cohort study on melanoma also found that combination with ipilimumab seems to have a higher objective response rate, PFS, and OS than monotherapy, but the rate of grade 3–5 toxicity is similar [39].

## 4. Limitations of CAR T-Cell Therapy in Solid Tumors

Chimeric antigen receptor (CAR) T cell therapy is an innovative tumor therapy that relies on cell surface antigen recombinant receptors to reactivate T lymphocytes for tumor resistance [40,41]. CAR-modified T cells not only obtain the properties of a “live drug” but also break through the constraints of traditional MHC receptors, enabling them to have long-term anti-tumor efficacy. Early clinical trials have shown that CD19 CAR-T cell therapy has a complete remission rate of up to 80% in acute lymphoblastic leukemia, significantly prolonging patient survival [42]. Notably, although transplanted T cells may be more potent than T cells from leukemia patients, it has the potential to mediate graft-versus-host disease [43]. Unfortunately, this success has not been replicated in solid tumors [44]. Solid tumors are denser in texture, have a high degree of heterogeneity in antigen expression, and are sequestered in organs or tissues. In addition, in the immunosuppressive microenvironment, it is difficult for T cell function to be at the most preferential activation level [45]. Therefore, the lack of a sufficient T cell infiltration and immunosuppressive microenvironment is the main obstacle for solid tumors to carry out CAR-T cell therapy [46]. Generally, in solid tumors, tumor-associated antigens are more accepted, such as EGFR, CEA, MUC1, etc. It is worth mentioning that these tumor-associated antigens also have lower expression in normal tissues [47,48]. Once tumor antigens lack specificity, off-target toxicity increases significantly [49,50]. For example, CRC patients treated with Her2-CAR-T cells developed severe off-target toxicity [51]. Therefore, tumor cell antigens that are differentially expressed from normal tissues must undergo rigorous evaluation and validation before they can be described as target antigens. In CRC, NKG2D, GUCY2C, and TAG-72 are promising target antigens [52,53,54]. A recent report pointed out that DCLK1-targeted CAR-T therapy has a significant effect on primary or metastatic CRC [55,56]. Surprisingly, a study indicated that bispecific Trop2/PDL1 CAR-T cells could significantly inhibit gastric cancer growth by intratumoral injection, and its inhibitory effect was more significant than that of Trop2-specific CAR-T cells [57]. In addition, CXCR2-expressing CAR-T cells were more sensitive to the IL-8-rich microenvironment in pancreatic cancer and had stronger anti-tumor activity against αvβ6-expressing pancreatic tumor xenografts [58]. Therefore, the combination of CAR-T cell therapy and ICIs seems to be more conducive to the tumor suppressor effect.

## 5. Immune-Related Adverse Events

**Figure 1:**
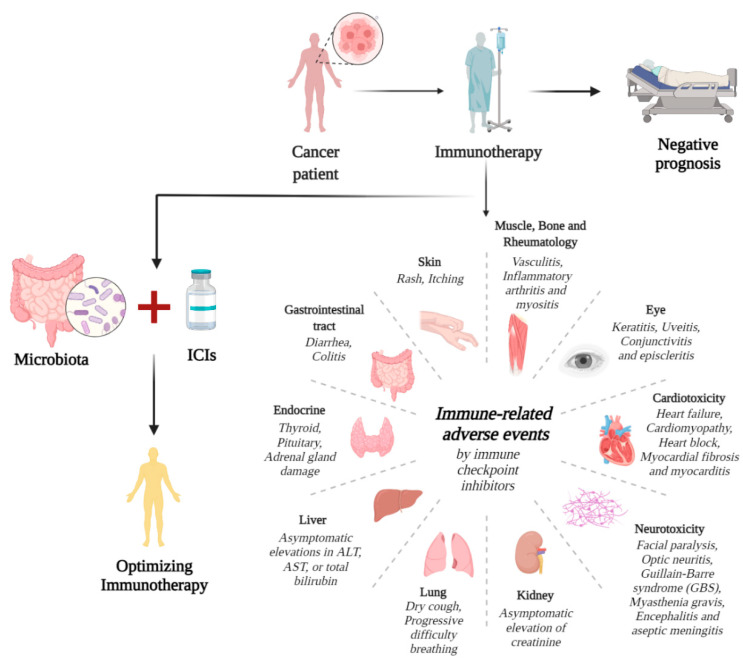
Immune-related adverse events of various organs or tissues caused by cancer immunotherapy. The immune adverse events mainly involved the skin, gastrointestinal tract, endocrine glands, liver, lungs, kidneys, nerves, heart, eyes, and musculoskeletal. The addition of the gut microbiota is expected to optimize the efficacy of cancer immunotherapy.

With the continuous promotion of ICIs in the clinic, immune-related adverse events have shown an exponential increase, which has become a major clinical challenge [59,60]. Although immune-related adverse events are not fatal, they often force patients and bedside physicians to make a clinical decision about whether to continue treatment. According to statistics, nivolumab induces endocrine toxicity, while pembrolizumab induces liver toxicity and joint pain [61]. Ipilimumab most commonly affects the skin and gastrointestinal tract and also has partial renal toxicity. A meta-analysis of the risk of ICIs supports that combination therapy (ipilimumab/nivolumab) is more likely to induce immune-related endocrine risk than monotherapy [62]. Another analysis added that combination therapy might even lead to discontinuation with high adverse effects [63]. However, appropriate combination regimens remain the primary consideration for clinicians compared with treatment-induced mortality. So far, the treatment of immune-related adverse events has mostly followed the empirical management of autoimmune diseases. Therefore, improving the complications of immunotherapy or exploring immune checkpoints with more therapeutic advantages are innovative measures. We briefly summarize partial immune-related adverse events and organizations and organs involved to provide reference to ICIs (Figure 1). With the advancement of sequencing, it was discovered that the intestinal microbiota is deeply involved in human metabolism and immunity, including tumors. Additionally, researchers are also gradually exploring how gut microbes reverse immune-related adverse events (Table 2).

## 6. Intestinal Microbiota Regulates Pharmacokinetics

Pharmacokinetics refers to the laws of drug absorption, distribution, metabolism, and excretion in the body [74]. Several studies have suggested that the gut microbiota may lead to changes in the chemical modification of medicinal products, resulting in activation, inactivation, or toxicity [75]. However, quantifying microbial intervention on pharmacokinetics remains challenging, especially based on the common basis of drug metabolism of the host and microbiota. Zimmermann M’s team combined the genetics of microbial symbiosis with gnotobiotics for the first time [76]. By monitoring the metabolic changes of brivudine (BRV) in mice caused by a single microbially encoded enzyme, they innovatively established a pharmacokinetic model and predicted quantitative microbial contributions to systemic drug metabolism [76]. They compared the serum kinetics of oral BRV in conventional (CV) and germ-free (GF) mice and demonstrated that BRV is converted to hepatotoxic bromoyluracil (BVU) by microbial enzymes, reducing BVU exposure in GF mice [76]. BVU interferes with pyrimidine metabolism in humans by binding to DPD, with lethal consequences in patients receiving chemotherapy with pyrimidine-like drugs such as 5-fluorouracil (5-FU) [77]. In addition, they found that Bacteroides and Oobacteria had the highest metabolic activity to convert BRV to BVU. This study provides the microbiome’s understanding of drug metabolism and aims to improve the response to drugs in chemotherapy patients. Additionally, they mapped human microbial drug metabolism by measuring the drug-modifying capacity of representative gut bacterial generations and systematically analyzing drug–microbe interactions by identifying drug-metabolizing microbial gene products [78]. Therefore, microbial intervention has a positive strategic effect on chemotherapy and drug metabolism.

## 7. Microbiota Correlates with Cancer Immunotherapy

The microbiota mainly inhabits the human intestines and maintains the host’s metabolism and immune crosstalk [79]. The microbial genome is more than 150 times that of the human body itself, so it is also called the second set of the human genome [80]. It covers a cumulative total of more than 1000 bacterial species, which endows the metagenomics with a common core while maintaining individual differences. Driven by the revolution of high-throughput sequencing technology, microbial research has quickly entered a new era of whole-genome sequencing from laboratory culture [81]. Metagenomics can also be combined with other omics to analyze the metabolism and immune processes of microorganisms, including transcriptomics, metabolomics, and proteomics [82,83]. Surprisingly, the intestinal microbiota has gradually been recognized as being able to intervene effectively in tumor immunotherapy, reduce the incidence of adverse events, and benefit patients [84].

In the early stage, in order to determine whether the microbiome interferes with the therapeutic activity of ICIs, melanoma mice (TAC mice with more severe tumors) carrying different intestinal microbiota but the same genotype from TAC and JAX were used to assess their correlation [85]. The results showed that Bifidobacterium was most significantly enriched in the feces of JAX mice and had a higher correlation with peripheral and intratumoral specific CD8+ T cell activation. Interestingly, after TAC mice were further transplanted or co-cultured with JAX mouse fecal bacteria, the differences between the two gradually approached [86]. This also directly established the status of the gut microbiome in immunotherapy. In the later stage, after PD-L1 ICIs were combined with mouse fecal suspension, tumor invasion and growth were consistently and significantly slowed, as expected [85].

## 8. Gut Microbiome Expands Path for Cancer Immunotherapy

In addition, Chaput et al. speculated from the baseline microbiome that the incidence of colitis in patients with metastatic melanoma treated with ipilimumab is regulated by the microbiome [87]. The study prospectively proposed that identifying the attributes of the microbiota is beneficial to avoid and control adverse risks that patients may encounter [87]. According to the RECIST 1.1 standard, patients with different microbiomes can be classified as responders (R) and non-responders (NR) [88]. On this basis, Derosa et al. successfully inoculated feces from R into NR through FMT technology to compensate for the anti-PD-1 immune effect [89,90]. Although the advantages of the microbiome in immunotherapy have become increasingly prominent, there are still challenges in screening out bacterial groups that are specifically enriched for ICIs [91]. With the support of Metatagenomic Shotgun Sequencing and Unbiased Metabolomic Profiling, stool analysis of R and NR is expected to provide clues for the identification of the intestinal microbiota [92].

Although the preclinical mouse model of intestinal microbiota intervention ICIs has been established, there is still a lack of evidence for its application to cancer patients. By analyzing the stool samples of melanoma patients treated with ICIs, Gopalakrishnan and others found significant differences in the diversity and number of intestinal microbiota between responders and non-responders [10]. In particular, the alpha diversity (a statistical method of the distribution and composition of bacteria in the sample) and the relative abundance of Ruminococcaceae (*p* < 0.01) in the feces of the responders were significantly increased, and the function of effector T cells in the tumor microenvironment (TME) was improved [10]. On the contrary, the feces of non-responders is often accompanied by a lower abundance of bacteria, and there is an enrichment of Bacteroidales, which block lymphatic infiltration and impair tumor immune response.

In an independent cohort study of combined treatment (ipilimumab plus nivolumab) of metastatic melanoma, Frankel et al. reported an increase in the abundance of Faecalibacterium in baseline stool samples of responders and non-responders [7]. The abundance of Faecalibacterium and the longer PFS showed a positive correlation trend [92]. According to the immunophenotype analysis of matched tumors and blood samples of patients in this cohort, the abundance of Faecalibacterium not only has a strong positive correlation with CD8 + T infiltration in the TME but also has a certain correlation with peripheral CD4+/CD8 + T cells [92]. Furthermore, in 32 NSCLC patients, enrichment of Enterococcus faecium also enhanced peripheral CD8+/CD4 + T cell responses and IFN-γ production, prolonging the PFS of R [93].

Coincidentally, by analyzing the stool samples from patients with metastatic melanoma, Matson et al. found that the degree of Bifidobacterium longum, Collinsella aerofaciens, and Enterococcus faecium enrichment in patients with anti-PD-1 was positively correlated with treatment response and inhibited tumor growth [94]. Additionally, after FMT rebuilds the intestinal environment of sterile mice, tumor control is significantly strengthened. This increased therapeutic effect is mediated by the activation of DCs, which leads to increased initiation and accumulation of CD8 + T cells in TME [95]. This also verifies that the intestinal microbiota from responders is involved in improving immunotherapy or as a candidate biomarker to promote immune surveillance of cancer [96]. Through 16S ribosomal RNA sequencing identification, Sivan et al. found that the effect of oral Bifidobacterium alone can be comparable to anti-PD-1 therapy, and the combined therapy can even completely inhibit tumors [85]. In addition, the metabolites of the microbiota not only reactivate dendritic cells (DCs) but also induce the metabolic reorganization of CD8 + T cells in the TME, providing a basis for the long-term survival of memory cells [97].

When comparing the intestinal microbiota of patients with different malignancies receiving immunotherapy, Routy et al. found that Akkermansia muciniphila (A. muciniphila) was more enriched in the feces of responders (*p* = 0.007) [90]. Interestingly, two independent teams studying NSCLC and renal cell carcinoma and studying metastatic melanoma also reached similar conclusions, suggesting the possibility of A. muciniphila as a biomarker [98,99]. It is worth mentioning that after co-culture of A. muciniphila with peripheral blood mononuclear cells of responders or non-responders, the effect of responders’ CD4+/CD8 + T cells in producing IFN-γ-related memory T cells was significantly stronger than that of non-responders [100]. At the same time, passing A. muciniphila through FMT can re-establish the immune checkpoint blocking effect of non-responders.

In addition, Bacteroidetes are increasingly recognized to be associated with the development and exacerbation of induced colitis [101,102]. In melanoma patients treated with ICIs, the abundance of Bacteroidetes appears to be positively correlated with colitis resistance [103]. Recently, a number of studies proved that different microbial populations excel in optimizing the therapeutic effects of different ICIs, improving the prognosis of patients [104,105]. We summarized the microbiota, tumors, and related immune regulation mechanisms involved in each study in Table 3.

In short, these studies broaden the perspective of the intestinal microbiota participating in cancer immunotherapy [106]. In fact, a variety of factors, such as diet, daily life, and drugs, are the basis for constructing the network of immune metabolism of the intestinal microbiota. Therefore, different life parameters may also affect the responsiveness of the intestinal microbiota in different populations and tumors.

**Table 3:** Intestinal microbiota optimizes cancer immunotherapy.

Microbial Species	Immune Optimization	Anti-PD-1/L1	Anti-CTLA-4	Tumors	References
*Alistipes putredinis*	Memory CD8+ T cells ↑ NK cells(peripheral) ↑	↑		NSCLC RCC	[100]
*Akkermansia muciniphila*	CXCR3+CCR9+CD4+ T cells ↑ DCs ↑ IL-12 ↑	↑		NSCLC RCC	[100]
*Bacteroides* spp.	MDSCs and Tregs ↑ Immune-related adverse events ↑ IL-12 ↓ DCs ↓	↓	↓	MM	[87]
*Bacteroides fragilis*	Th1 cells ↑ Foxp3+ Tregs ↑ DCs ↑		↑	MM NSCLC	[107]
*Bifidobacterium* spp.	DCs ↑ Lymphocytes ↑ IFN-γ ↑ Pro-inflammatory cytokine ↑ Tumor-specific CD8+ T cells ↑	↑		MM	[94] [85]
*Enterococcus faecium*	T cell responses ↑	↑		MM	[10]
*Escherichia* *Clostridium*	Differentiation of Tregs ↑ Inflammation ↓		↑	MM	[7]
*Faecalibacterium.* spp.	CD4+/CD8+ T cells ↑ Tregs ↑ ICOS expression of T cells ↑	↑	↑	MM	[87]
*Ruminococcaceae* spp.	Antigen presentation ↑ T cells ↑ IFN-γ CD8+ T cells ↑	↓		MM	[94]
Microbial-derived SCFAs (butyrate, propionate)	Differentiation of Tregs ↑		↑	CRC	[108]

## 9. The Clinical Application of Intestinal Microbiota

**Figure 2:**
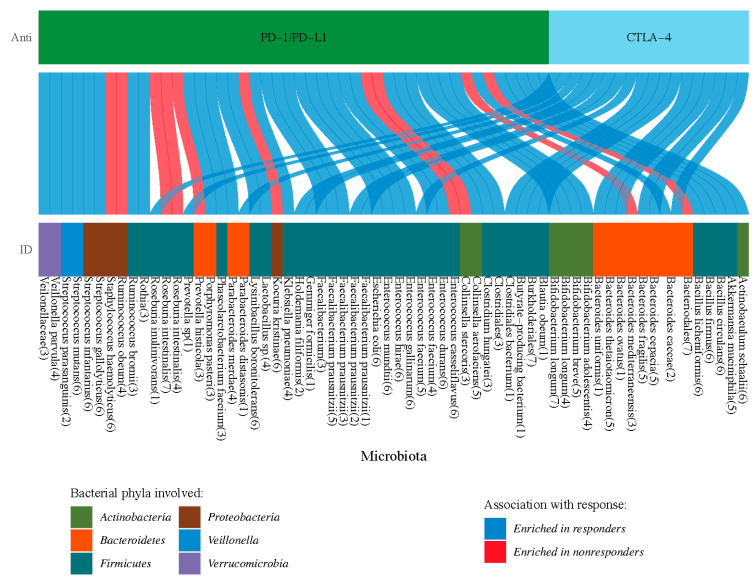
Differences in intestinal microbiota enrichment in ICIs (anti-PD-1/PD-L1 or anti-CTLA-4). Sankey diagrams provide visual clues to the enrichment characteristics of microorganisms in different ICIs. Blue band: the bacterial microbiota enriched in patients responding to ICIs, including Akkermansia muciniophila, Bifidobacterium species and Bacillus species, etc. Red band: the bacterial microbiota enriched in patients who did not respond to ICIs, including Staphylococcus haemolyticus, Bacteriodales, and Prevotella histicola, among others. The number in brackets indicates the source of the reference. (1) [87] (2) [92] (3) [10] (4) [94] (5) [100] (6) [90] (7) [109].

With the continuous expansion of research data, the gut microbiota provides unprecedented opportunities for the development of immunotherapy-related therapies. Although the mechanism of the gut microbiota in cancer immunotherapy remains to be explored, many cutting-edge studies provide valuable clinical evidence for the gut microbiota with application potential. According to recent clinical studies, we use a Sankey diagram to build a very meaningful bridge between ICIs (anti-PD-1/PD-L1 or anti-CTLA-4) and the enrichment of the intestinal microbiota (Figure 2).

Intestinal microbiota optimization and adjuvant immunotherapy have been confirmed, which provides confidence in reducing the complications of cancer immunotherapy [110]. Among them, the most common immune-related adverse event is related to colitis, but the cause is still unknown. Interestingly, colitis was effectively relieved under the intervention of the lactic acid bacteria Lactobacillus reuteri, and the patient’s weight loss and inflammation symptoms were significantly improved [111]. In addition, the activation of the protection of Lactobacillus reuteri may be based on the decrease in lymphocyte distribution [112]. The emergence of tumor microecological immunonutrition further provides an opportunity for the microbiota as a breakthrough in cancer immunotherapy [113]. The ketogenic diet has also been reported to enhance the efficacy of immunotherapy [114].

A number of studies have proposed that adjusting the composition of the gut microbiota can effectively improve immunotherapy, including FMT, dietary intervention, probiotics, or prebiotics [115]. Among them, FMT is gradually recognized as a beneficial clinical intervention. FMT is perfused or orally transplanted into the patient’s intestines in the form of bacterial liquid or capsules [116]. At present, FMT has been proven to be the first-line treatment for relapsed/refractory Clostridium difficile infection (CDI) and has been included in the national clinical guidelines [117]. Since microbial research turns to an in-depth mechanism direction, the role of the microbiota and metabolites in the field of inflammatory bowel disease and cancer will undoubtedly be further highlighted [118,119,120]. Certainly, FMT is in its infancy in cancer clinical exploration, but it is a landmark discovery in the field of cancer therapy.

## 10. The Combination of FMT and Immunotherapy

The combination therapy of FMT and ICIs not only takes into account the curative effect but also guarantees better safety. Baruch et al. performed intestinal microbiota reconstruction in 10 groups of patients with anti-PD-1 refractory metastatic melanoma [121]. After using vancomycin to destroy their own intestinal microbiota for 3 days, the patient rebuilt a new intestinal environment through FMT [122]. After receiving the anti-PD-1 ICIs again, two patients had partial remission, and one patient had complete remission with PFS for more than 6 months. Only some patients experienced discomfort, such as abdominal distension, and no other adverse reactions occurred [123].

Another study also performed FMT and PD-1 monoclonal antibody combination therapy on 15 patients with anti-PD-1 resistant metastatic melanoma [124]. The results showed that six patients responded to anti-PD-1, and only three patients experienced grade 3 adverse events. The study also further clarified that the combination of FMT and anti-PD-1 therapy weakened bone marrow-induced immune suppression, activated CD56 + CD8 + T cells, and downregulated the expression of IL-8 [125]. In contrast, patients with poor intestinal microbiota exhibited weak anti-tumor immunity due to restricted antigen presentation. The latest FMT and immunotherapy-related clinical trials provide a basis for clarifying the involvement of intestinal microbiota in the regulation of ICIs (Table 4). The clinical trial (NCT03353402) attempts to improve the gut microbiota of melanoma patients who cannot benefit from immunotherapy through FMT. Another clinical trial (NCT05008861) tried to rebuild the gut microbiota in the immunotherapy of non-small cell lung cancer through FMT. The combination of FMT and ICIs will make a greater contribution to the prognosis of patients. In addition, before the combination therapy of FMT and ICIs, the patient must consume antibiotics orally to destroy the harmful intestinal microbiota in the body. According to reports, the vancomycin–neomycin regimen is currently the most effective preparation for treatment [126]. The results of clinical trials and conventional treatment indicate that antibiotic exposure may reduce the OS rate of patients receiving immunotherapy, but the potential intervention is not yet clear [12,13].

## 11. Probiotics and Prebiotics

Undoubtedly, FMT is a highly potential microbial intervention therapy. However, before clinical application, pathogen screening is indispensable to prevent the induction of bacteremia. Probiotics and prebiotics have received widespread attention due to their low price and easy access [127]. As a biological response modifier, probiotics are involved in the competitive displacement of pathogens, the assembly of antiviral proteins, and the maintenance of physiological functions of the epithelial–mucosal barrier [128]. Probiotics not only directly regulate immune and metabolic processes through intestinal immune cells and epithelial cells but also indirectly maintain the immune state of multiple sites by regulating intestinal microbiota [129]. Probiotics, such as Lactobacillus and Bifidobacterium, can promote antigen-specific recognition of infected monocytes to cut off the transmission route [28,130]. A clinical trial involving 20 CRC patients (NCT03072641) revealed that bifidobacteria and lactobacilli altered the immune responsiveness of the intestinal mucosa compared with baseline tissue or stool samples [131]. Further, the clinical trial also found changes in the expression of cytokines, such as IL-10, IL-12, IL-17, etc., in the mucosa [131]. Coincidentally, probiotic preparations containing Lactobacillus casei have significantly improved in ensuring surgical resection and recurrence-free survival of bladder cancer [132]. In addition, there is evidence that prebiotics such as inulin, lactulose, and galactooligosaccharides can selectively induce the proliferation of bifidobacteria and lactobacilli [133,134]. However, due to a lack of scientifically rigorous evidence, most probiotics and prebiotics are still not included in the drug category. The initial success of probiotics in CDI and inflammatory bowel disease has given strong confidence and spurred a focus on providing strong scientific evidence for their efficacy [135]. It cannot be ignored that the fraction of the symbiotic microbiota with lower individual variability is of great significance in manipulating host physiology. Tanoue et al. obtained 11 fecal microbiota derived from healthy people and found that they strongly induced interferon γ + CD8 T cells [136]. Further colonizing the microbiota in mice, they found that the mixed microbiota not only enhanced the anti-tumor immune effect of ICIs but also effectively avoided immune-related colitis [136]. Therefore, the research on the microbiome will provide a powerful boost to the progress of clinical tumors and other multidisciplinary subjects.

## 12. Antibiotic and Immunotherapy Efficacy

For immunotherapy patients, the use of antibiotics should be carefully weighed. Exposure to broad-spectrum antibiotics, even when the source of infection is identified, can still impair gut microbiota homeostasis in cancer patients receiving immunotherapy [137]. One study showed that, regardless of whether antibiotics were used before and after anti-PD-1, the median survival rate of patients was only half that of those who did not receive antibiotic exposure [138]. A multicenter, prospective cohort study of 196 samples (NSCLC, melanoma, RCC, and head and neck cancer) also supported that antibiotics reduce PD-1/PD-L1 response to treatment, and patients have lower OS [139]. However, a retrospective study of 74 NSCLC patients treated with nivolumab did not find significant differences in efficacy or PFS [140]. Although the impact of antibiotics on immunotherapy remains controversial, it is undeniable that clinicians need to carefully consider the duration and dose of antibiotic exposure. We look forward to more prospective studies being conducted to clarify this issue.

## 13. Conclusions

The intestinal microbiota and immunotherapy are a new cross-cutting field. Microbiota association research and avant-garde sequencing technologies have shifted from descriptive macroscopic research to the regulation of the immune metabolism of specific microbiota. Through intervention modes such as FMT, the microbiota can not only reduce immune-related adverse events but also serve as a biomarker to predict the prognosis of patients. Based on clinical trials and bioinformatics data, the development of accurate and effective individualized microbial therapy will be the direction of tumor treatment in the future.

## Figures and Tables

**Table 1:** Some solid tumors receiving immunotherapy.

ICIs		Cancer	Results	References
Anti-PD-1	Nivolumab	Advanced cervical cancer	36% patients had stable disease (9/25; 90% CI, 20.2–54.4%) for a median of 5.7 months. Estimated PFS and OS at 6 months were 16% and 78.4%.	[23]
	Pembrolizumab	NSCLC	Half-year PFS:22%; median PFS:2.8 months (95% CI: 1.5–4.1); median OS: 11.7 months (95% CI: 7.6–13.4).	[24]
	Cemiplimab	Advanced squamous cell carcinomas	PD-L1 expression was ≥1% in 18% patients and <1% in 11% of patients.	[25]
Anti-PD-L1	Atezolizumab	Advanced triple-negative breast cancer	Median survival to progression and overall survival were 5.5 months (95% CI, 5.1–7.7 months) and 14.7 months (95% CI, 10.1, not evaluable).	[26]
	Avelumab	Advanced Merkel cell cancer	ORR:48.0%; median duration of treatment:7.4 months (1.0–41.7 months).	[27]
	Durvalumab	NSCLC	Median PFS:17.5 months (95% CI, 13.2–24.9); median OS:47 months (95%CI, 47 [NR]).	[28]
Anti-CTLA4	Ipilimumab	Metastatic melanoma	Survival rates at 5 years in patients were OS 11%.	[29]
Combination	Ipilimumab + nivolumab	Metastatic CRC	PFS:76% (9 months) and 71% (12 months); respective OS: 87% and 85%.	[30]

**Table 2:** Common immune-related adverse events.

Systemic or Tissue Toxicity	Clinical Manifestation	Treatment Measures	References
Skin	Rash, itching	Symptomatic treatment (topical corticosteroids and oral antihistamines).	[64]
Gastrointestinal tract	Diarrhea, colitis	Rehydrate, rule out infection, and administer oral or intravenous corticosteroids. Colonoscopy or sigmoidoscopy.	[65]
Endocrine	Thyroid, pituitary, or adrenal gland damage	During ICIs, thyroid function is regularly monitored.	[66]
Liver	Asymptomatic elevations in ALT, AST, or total bilirubin	With oral corticosteroids, immune-mediated hepatitis usually resolves within 4–6 weeks.	[67]
Lung	Dry cough, progressive difficulty breathing	Nearly 75% of patients may require discontinuation of ICIs.	[68]
Kidney	Asymptomatic elevation of creatinine	Corticosteroid therapy and sparing immunotherapy are recommended. Renal biopsy is necessary for higher-grade events.	[69]
Neurotoxicity	Facial paralysis, optic neuritis, Guillain-Barre syndrome, myasthenia gravis, encephalitis, and aseptic meningitis	Steroid therapy is used to relieve mild symptoms, but severe toxicity requires high doses or other therapies.	[70]
Cardiotoxicity	Heart failure, cardiomyopathy, heart block, myocardial fibrosis, and myocarditis	ICIs were discontinued, and steroid therapy was initiated.	[71]
Eye	Keratitis, uveitis, conjunctivitis, and episcleritis	Topical or systemic corticosteroid therapy.	[72]
Muscle, Bone and Rheumatology	Vasculitis, inflammatory arthritis, and myositis	Low-dose steroids have some effects.	[73]

**Table 4:** Related clinical trials of FMT in cancer immunotherapy.

NCT Number	Title	Status	Conditions	Interventions	Phases
NCT05008861	Gut Microbiota Reconstruction for NSCLC Immunotherapy	Not yet recruiting	Non-Small Cell Lung Cancer	Procedure: Capsulized Fecal Microbiota Transplant Drug: Anti-PD-1/PD-L1 Drug: Platinum-based chemotherapy	Phase 1
NCT04924374	Microbiota Transplant in Advanced Lung Cancer Treated with Immunotherapy	Recruiting	Lung Cancer	Dietary Supplement: Microbiota Transplant plus anti-PD-1 therapy Drug: anti-PD-1 therapy	Not Applicable
NCT04729322	Fecal Microbiota Transplant and Re-introduction of Anti-PD-1 Therapy (Pembrolizumab or Nivolumab) for the Treatment of Metastatic Colorectal Cancer in Anti-PD-1 Non-responders	Recruiting	Metastatic Colorectal Adenocarcinoma Metastatic Small Intestinal Adenocarcinoma Stage IV Colorectal Cancer	Procedure: Fecal Microbiota Transplantation Drug: Metronidazole Biological: Nivolumab/Pembrolizumab	Early Phase 1
NCT03353402	Fecal Microbiota Transplantation (FMT) in Metastatic Melanoma Patients Who Failed Immunotherapy	Recruiting	Melanoma Stage Iv Unresectable Stage III Melanoma	Procedure: Fecal Microbiota Transplant (FMT)	Phase 1
NCT03772899	Fecal Microbial Transplantation in Combination with Immunotherapy in Melanoma Patients (MIMic)	Recruiting	Melanoma	Drug: Fecal Microbial Transplantation	Phase 1
NCT04758507	Fecal Microbiota Transplantation to Improve Efficacy of Immune Checkpoint Inhibitors in Renal Cell Carcinoma	Recruiting	Renal Cell Carcinoma	Biological: donor FMT Other: Placebo FMT	Phase 1 Phase 2

## Abbreviations

Colorectal cancer: CRC; cytotoxic T lymphocyte antigen 4: CTLA-4; dendritic cells: DCs; fecal microbial transplantation: FMT; high microsatellite instability: MSI-H; immune checkpoint inhibitors: ICIs; Microsatellite Stabilization: MSS; metastatic triple-negative breast cancer: mTNBC; non-responders: NR; non-small cell lung cancer: NSCLC; overall survival: OS; programmed death receptor 1/programmed death ligand 1: PD-1/PD-L1; progression-free survival: PFS; tumor microenvironment: TME; tumor mutation burden: TMB.
